# Recurrent skull vault actinomycosis: A case report and review of literature

**DOI:** 10.1016/j.idcr.2021.e01215

**Published:** 2021-07-03

**Authors:** Abdur Rehman Mohamad, Junais Koleri, Hussain Mohamed Sultan Hussain, Hussam Al Soub, Muna Al Maslamani

**Affiliations:** a Department of Infectious Diseases, Communicable Diseases Centre, Hamad Medical Corporation, Qatar; b Department of Neurosurgery, Hamad Medical Corporation, Qatar

**Keywords:** Actinomycosis, Calvarial infection, Recurrent, Osteomyelitis

## Abstract

Actinomycosis is an uncommon cause of central nervous system infection. A case of skull bone osteomyelitis with epidural empyema is presented. A 44-year-old man presented with chronic osteomyelitis of skull vault with epidural and subgaleal collection diagnosed by histopathology as actinomycosis. He had similar lesion at the same site 10 years ago, which was excised completely. Recurrent Actinomycosis of the skull vault is uncommon in literature. This case highlights the importance of considering actinomycosis as a differential diagnosis of tumorous growths and stresses on the importance of tissue histopathology for diagnosis and need for surgery to control the disease. Treatment is prolonged, therefore compliance with the long-term antibiotic duration is essential to prevent complications and avoid recurrence.

## Introduction

Actinomycosis is a subacute bacterial infection characterized by tissue fibrosis, abscess formation and draining sinuses. *Actinomyces* species are normal inhabitants of the oral cavity, the gastrointestinal and pelvic tracts and are not found existing freely in nature. Cervicofacial, thoracic and abdominal are the most common forms of actinomycosis. Central nervous system (CNS) actinomycosis are rare and usually result from direct extension of a neighboring focus or from hematogenous spread from a distant focus. Brain abscess are the most reported CNS manifestations. Calvarial and epidural abscess are extremely rare, and few cases have been reported in the literature. We herein, present a case of recurrent frontal skull bone osteomyelitis with epidural subgaleal collections caused by actinomycosis and review the literature.

## Case history

A 44-year-old Sudanese man with no significant comorbidities was referred to neurosurgery outpatient clinic with the complaint of left frontoparietal scalp mass lesion, progressively increasing in size over 1 year. Patient did not seek medical advice until he started to experience unremitting hemi-cranial headache and cosmetic disturbance. He did not have fever or seizures. There was no history of trauma or dental problems.

On examination he appeared healthy and not in distress, with normal vital signs. He was alert oriented, and cranial nerves were intact. There was no motor or sensory or cerebellar function deficit. Ear, nose, mouth, and throat examinations did not show any focus of infection. He never smoked or consumed alcohol. He has been working as a Shepherd for more than 20 years.

His past history was remarkable for a similar lesion in the same location 10 years ago. The lesion appeared spontaneously over a period of 6 months. There was no antecedent history of trauma or surgery in the scalp or oro-maxillo-facial area. He had no history of head, neck, or oral infections. CT scan of head at that time showed left frontal mass of 4.5 cm width, homogeneous soft tissue mass, which appeared adherent to the underlying bone with a well-defined erosion confined to the outer table with medullary sclerosis and intact inner table. His routine laboratory tests at that time were within normal limits. No evidence of immunosuppression was reported. The mass was excised, and histopathology revealed the presence of micro abscesses with vague granuloma formation surrounding filamentous bacteria consistent with the diagnosis of actinomycosis. Oral amoxicillin was started, and he was asked to follow up in infectious diseases clinic; however, he was not compliant to his treatment and did not follow up. In the current presentation, his initial work up showed Hemoglobin 15.5 g/dl, White Blood Cell count 9500/microL (N93 %, L 5%, E 0.1 %), C Reactive Protein 12 mg/dL, procalcitonin 0.03 ng/mL. HIV test non-reactive, and normal blood glucose, renal and liver functions.

**Fig. 1:**
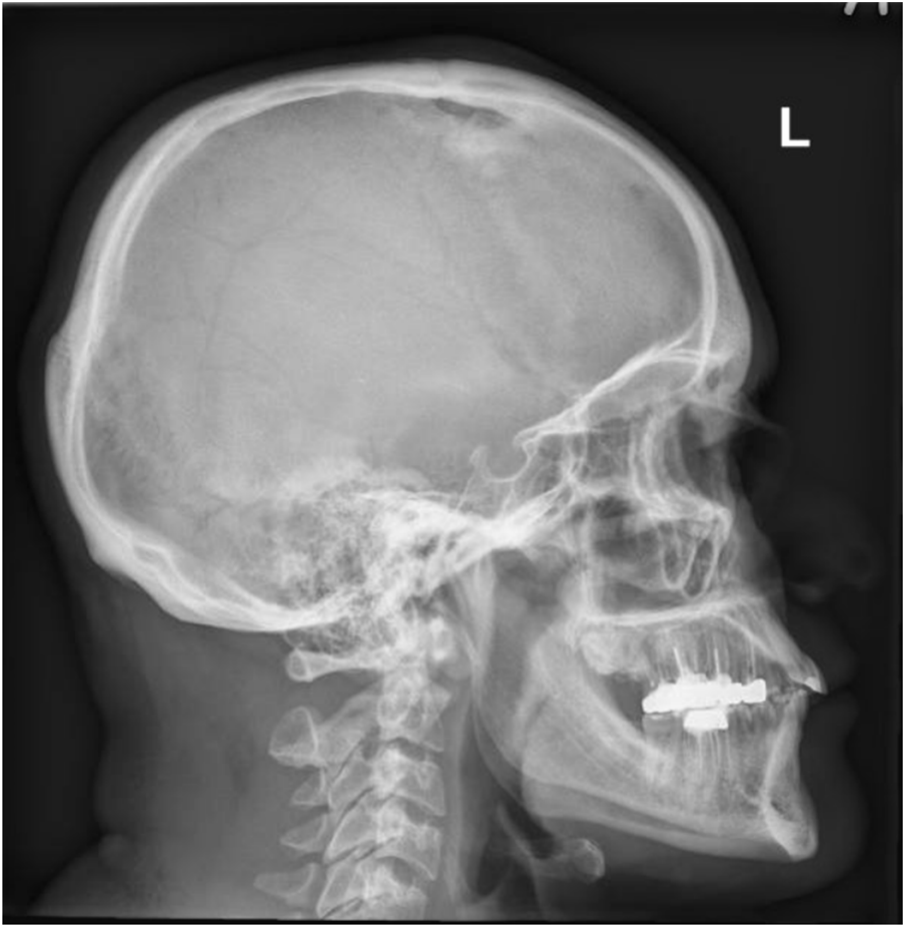
Skull x-ray showing osteolytic lesion.

**Fig. 2:**
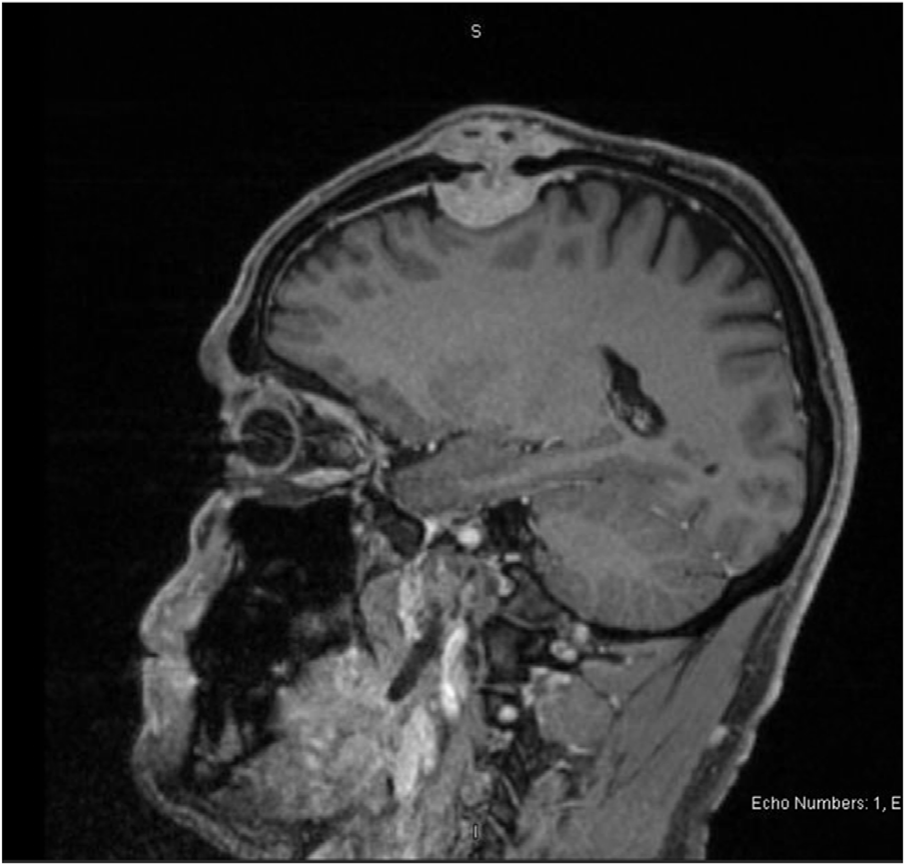
MRI- Sagittal plane. MRI shows a well defined rounded extra-axial lesion seen in the left frontal area measuring 32 × 26 × 18.5 mm in AP transverse and craniocaudal dimension, it is connected to a superficial subcutaneous scalp soft tissue swelling showing the same characteristics and measuring about 37 × 30 mm in transverse and craniocaudal dimension.

**Fig. 3:**
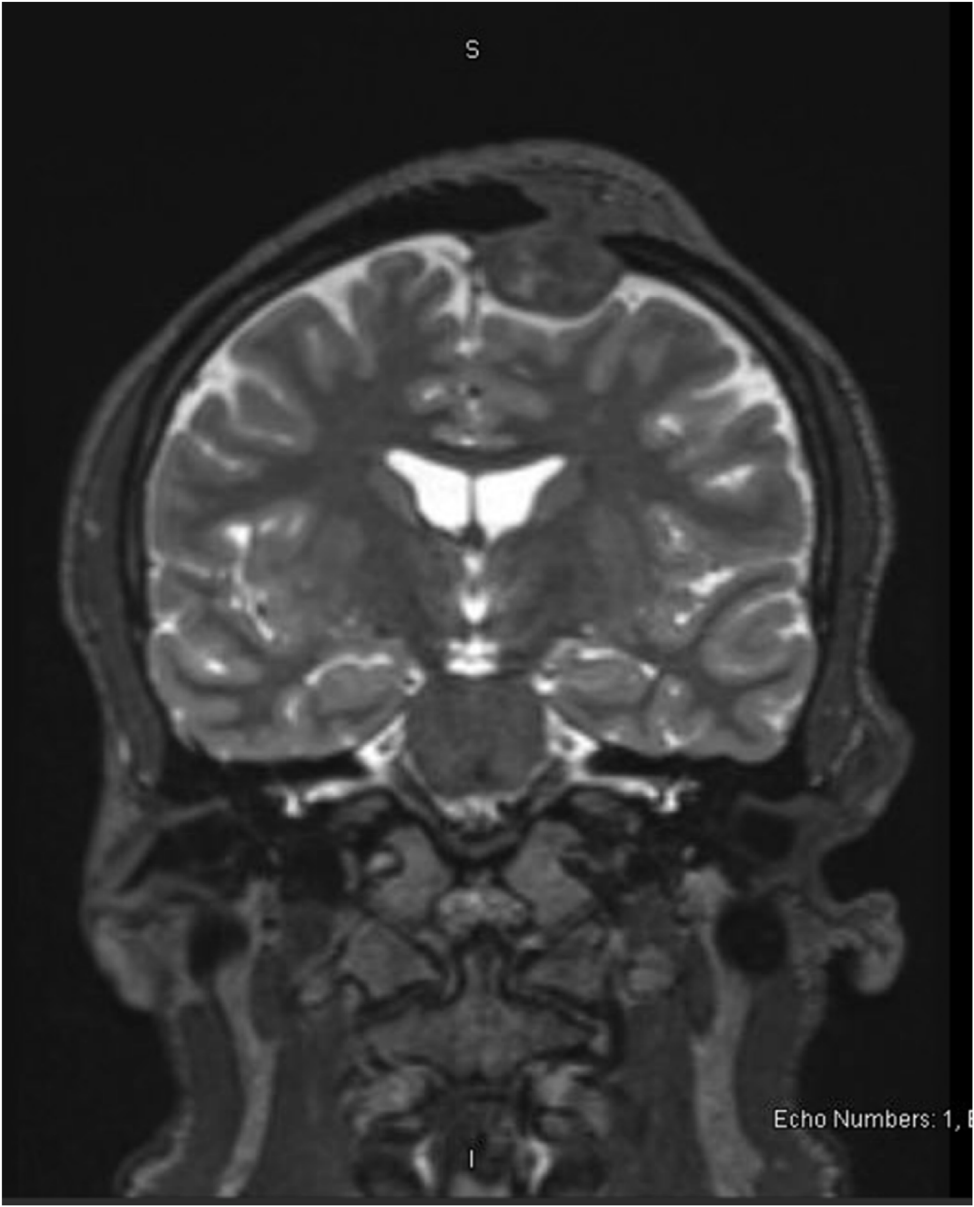
MRI- coronal plane.

**Fig. 4:**
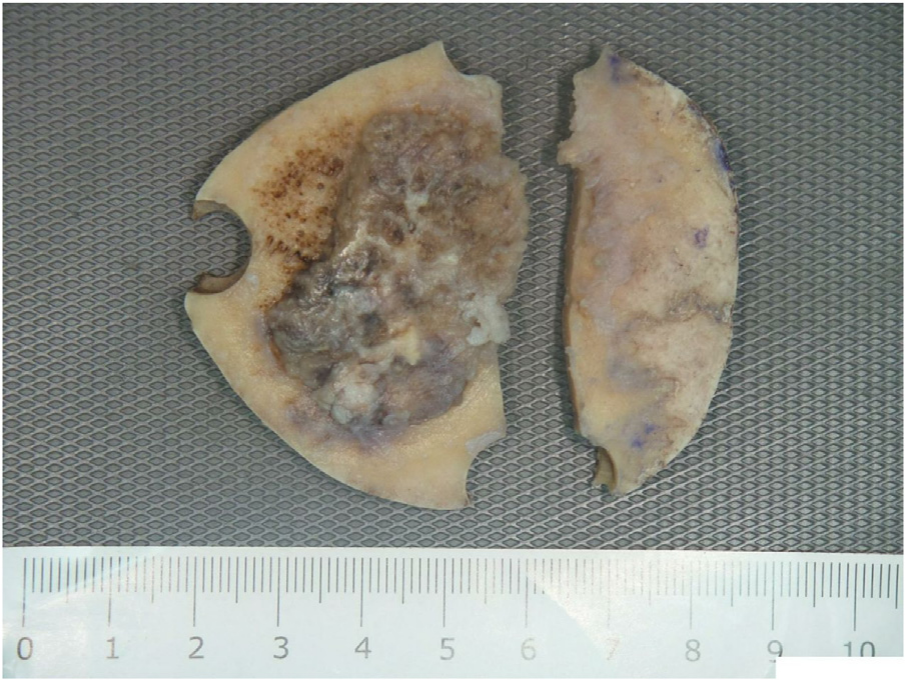
Cut section of skull showing irregular bosselated grey-brown destructive lesion with purulent foci.

**Fig. 5:**
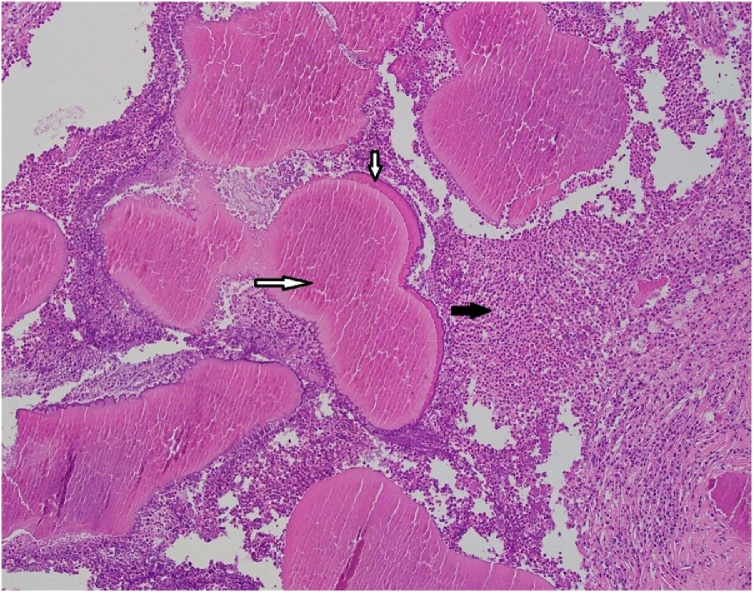
Gram stain. Multiple colonies of radiating filamentous organisms (horizontal white arrow) with peripheral rim of splendor hoeppli phenomenon (vertical white arrow) surrounded by dense neutrophilic infiltrate (black arrow).

Skull x-ray (Fig. 1) revealed osteolytic lesion in the left frontal bone with sclerotic margins and increased thickness of the skull. MRI (Fig. 2, Fig. 3) showed well-defined rounded extra-axial lesion seen in the left frontal area measuring 32 × 26 × 18.5 mm, connected to a superficial subcutaneous scalp soft tissue measuring about 37 × 30 mm. Brain parenchyma was unaffected. Craniotomy and microsurgical resection of the lesion was done. Intraoperatively, with opening over the previous scar, a semi-rounded mass, firm to hard in consistency, with surrounding cheesy material lesion (Fig. 4), invading the bone, with dural infiltration but no intra-axial extension. The mass was excised completely. Excision biopsy histopathology revealed numerous Actinomyces organisms positive for Gomori Methenamine-Silver Nitrate (GMS) Stain, Periodic acid‑Schiff (PAS) and Period acid stain with diastase (PASD), surrounded by well-defined granulomas and micro-abscesses (Fig. 5). Patient had an uneventful post-operative period and was discharged on the 4th post-operative day. Ceftriaxone was started for 2 weeks with a plan to continue with oral amoxicillin-clavulanate for 6 more months with close follow up.

## Discussion

Types of CNS actinomycotic lesions include brain abscess, meningitis, subdural empyema, actinomycoma, and epidural abscess; with brain abscess accounting for 75 % of the cases [1]. CNS involvement occur either via hematogenous dissemination or due to direct spread of infection from cervicofacial actinomycosis or following trauma [2]. Skull base and temporal bone osteomyelitis also have been reported, secondary to spread of infection from the oral cavity. However, epidural infection and calvarial osteomyelitis are extremely rare. Literature search revealed 5 reports of isolated calvarial osteomyelitis which are summarized below.

### Case 1

13 years old Indian boy presented with healing vertex ulcer, seizure and features of raised intracranial pressure [3]. CT imaging showed chronic osteomyelitis of frontal vault with underlying epidural mass causing midline shift. Scalp lesion biopsy proved actinomycosis. He was treated with intravenous crystalline penicillin and oral co-trimoxazole for 6 weeks followed by cotrimoxazole and erythromycin for 6 months. He recovered well, however epidural mass and skull thickening persisted.

### Case 2

40 years old Spanish-American was admitted in 1967 with left hemiparesis and recurrent generalized seizures [4]. His past history was significant for purulent discharge from a cutaneous sinus from left side of mandible several years ago, treated with surgery and penicillin injection. His skull x-ray showed uniform extensive thickening of left lateral skull vault involving frontal bone, parietal bone sphenoid and walls of orbit, with involvement of middle cranial fossa. Upon craniectomy, thick tenacious material was found in the epidural space, biopsy, and culture of which established actinomycosis. He was treated with prolonged course of penicillin therapy. He improved clinically; however, repeat skull films obtained after 2 years did not show appreciable difference.

### Case 3

35 years old Indian woman admitted with left hemiparesis following few months of headache and scalp swelling [5]. CT scan demonstrated hyperdense lesion in the right parietal bone along with dural thickening. Subsequent MRI further showed T1 isointense, T2 hypointense lesion in the epidural space infiltrating into the brain parenchyma causing midline shift. There was also and extracranial component which was connected to the intracranial component. Biopsy was obtained from the lesion, histopathology of which confirmed actinomycosis. She was treated with penicillin. Follow-up MRI done 5 months later showed residual lesion of smaller size. A second follow-up MRI done 1.5 years after surgery showed persistence and mild increase in the size of the lesion compared with the second MRI. She was continued on antimicrobial therapy.

### Case 4

21 years old Irish man presented with left frontal scalp abscess [6]. Incision and drainage were done, and he was discharged on oral amoxicillin-clavulanate. Four months later he came with persistent discharge from the same site. This time CT scan was done which revealed osteomyelitis, full thickness bone erosion and pus in the underlying frontal sinus. Craniotomy and drainage were done, and pus culture subsequently grew actinomycosis. He was commenced on long term antibiotic therapy. He remained systemically well.

### Case 5

A case of left parietal calvarial osteomyelitis due to actinomycosis in a 32 years old previously healthy woman is described [7]. She presented in the puerperal period. There was no extracranial focus. She responded well to a combination of surgery and antibiotics.

Our case is unique for both the site of occurrence and the relapse after 11 years. In the first episode in 2010 he had osteomyelitis involvement of only the outer table of calvarium. Adequate surgical excision was done, however, he did not comply with antimicrobial therapy and lost from follow up. The second episode was in the same site which suggests that it was a relapse rather than reinfection. This time lesion penetrated the inner table with extension to epidural space. Fortunately, there was no brain parenchymal involvement. No other focus of involvement with actinomycosis infection was identified in our patient.

In the case series reported by Smego et al., out of 70 patient with CNS actinomycosis only one patient had relapse [1]. This patient had temporal lobe abscess treated with needle aspiration followed by antibiotics, duration was not specified. He had relapse of actinomycosis as multiple brain abscess after 29 months. Relapse of actinomycosis have been described in many scenarios, all due to inadequate duration of antimicrobial therapy [[8], [9], [10], [11]]. Review of the cases reported before and our case indicate that Actinomycosis of the skull is a disease of young, immunocompetent patients. The presentation is usually insidious. Intracranial extension is common with some patients presenting with neurologic deficits. Imaging especially CT scan or MRI are very valuable to identify the lesion, however culture and histopathology are necessary to confirm the diagnosis. The mainstay of management for actinomycosis is medical therapy [12], with surgical drainage indicated if the collection is large or to establish a diagnosis. Penicillin is the drug of choice. Patients with actinomycosis require prolonged (6- to 12-month) antimicrobial treatment with high doses of penicillin G or amoxicillin. The duration of antimicrobial therapy could be reduced for patients in whom optimal surgical resection has been performed [2]. The outcome is in general favorable; however, relapse is not uncommon reflecting the need to educate patients for the need of prolonged antibiotics therapy and the importance of adherence to treatment.

## Conclusion

In conclusion, Actinomycosis is chronic indolent locally spreading infection caused mainly by breach of mucosa. We present a rare case of actinomycosis of frontal skull bone which recurred 11years after successful surgery. Keeping a high index of suspicion is essential to avoid delayed diagnosis and to start appropriate treatment. This case demonstrates that prolonged antibacterial treatment is of paramount importance to prevent relapse even after achieving adequate surgical excision.

## Declaration of Competing Interest

None.

## References

[1] Smego R.A. (1987). Actinomycosis of the central nervous system. Rev Infect Dis.

[2] Valour F., Sénéchal A., Dupieux C., Karsenty J., Lustig S., Breton P. (2014). Actinomycosis: etiology, clinical features, diagnosis, treatment, and management. Infect Drug Resist.

[3] Narayan S.K., Swaroop A., Jayanthi S. (2009). Chronic epidural intracranial actinomycosis: a rare case. Ann Indian Acad Neurol.

[4] Kirsch W.M., Stears J.C. (1970). Actinomycotic osteomyelitis of the skull and epidural space: case report. J Neurosurg.

[5] Kramadhary H., Nagarajan K., Ramesh A., Gochhait D. (2019). Transcalvarial and transdural involvement of skull actinomycosis with recurrence. Indian J Neurosurg.

[6] Nugent N.F., Murphy M., Kelly J. (2010). Scalp abscess – a cautionary tale. J Plast Reconstr Aesthet Surg.

[7] Roopesh Kumar V.R., Madhugiri V.S., Gundamaneni S.K., Verma S.K. (2014). Actinomycotic osteomyelitis of the cranial vault presenting with headache: an unusual presentation. BMJ Case Rep [Internet].

[8] Padmanabhan A., Thomas A.V. (2017). Recurrent endobronchial actinomycosis following an interventional procedure. Lung India Off Organ Indian Chest Soc.

[9] Fry R.D., Birnbaum E.H., Lacey D.L. (1992). Actinomyces as a cause of recurrent perianal fistula in the immunocompromised patient. Surgery.

[10] Coakham H.B., Ashby E.C. (1972). Actinomycosis in recurrent psoas abscess. Proc R Soc Med.

[11] Rajput V., Warad B., Chincholkar V., Davane M., Nagoba B. (2018). Recurrent abdominal actinomycosis with multiple organ involvement: a rare clinical presentation. Acta Med Iran.

[12] Brook I. (2008). Actinomycosis: diagnosis and management. South Med J..

